# BioSamples database: FAIRer samples metadata to accelerate research data management

**DOI:** 10.1093/nar/gkab1046

**Published:** 2021-11-08

**Authors:** Mélanie Courtot, Dipayan Gupta, Isuru Liyanage, Fuqi Xu, Tony Burdett

**Affiliations:** European Molecular Biology Laboratory, European Bioinformatics Institute, Wellcome Genome Campus, Hinxton, UK; European Molecular Biology Laboratory, European Bioinformatics Institute, Wellcome Genome Campus, Hinxton, UK; European Molecular Biology Laboratory, European Bioinformatics Institute, Wellcome Genome Campus, Hinxton, UK; European Molecular Biology Laboratory, European Bioinformatics Institute, Wellcome Genome Campus, Hinxton, UK; European Molecular Biology Laboratory, European Bioinformatics Institute, Wellcome Genome Campus, Hinxton, UK

## Abstract

The BioSamples database at EMBL-EBI is the central institutional repository for sample metadata storage and connection to EMBL-EBI archives and other resources. The technical improvements to our infrastructure described in our last update have enabled us to scale and accommodate an increasing number of communities, resulting in a higher number of submissions and more heterogeneous data. The BioSamples database now has a valuable set of features and processes to improve data quality in BioSamples, and in particular enriching metadata content and following FAIR principles. In this manuscript, we describe how BioSamples in 2021 handles requirements from our community of users through exemplar use cases: increased findability of samples and improved data management practices support the goals of the ReSOLUTE project, how the plant community benefits from being able to link genotypic to phenotypic information, and we highlight how cumulatively those improvements contribute to more complex multi-omics data integration supporting COVID-19 research. Finally, we present underlying technical features used as pillars throughout those use cases and how they are reused for expanded engagement with communities such as FAIRplus and the Global Alliance for Genomics and Health. Availability: The BioSamples database is freely available at http://www.ebi.ac.uk/biosamples. Content is distributed under the EMBL-EBI Terms of Use available at https://www.ebi.ac.uk/about/terms-of-use. The BioSamples code is available at https://github.com/EBIBioSamples/biosamples-v4 and distributed under the Apache 2.0 license.

## INTRODUCTION

The EMBL-EBI BioSamples database provides a unique reference for samples metadata across EMBL-EBI archives, the International Nucleotide Sequence Database Collaboration (INSDC) (1), and beyond. It enables centralised representation of samples and their description, as well as relationships between samples and assays across repositories and linkage back to samples’ donors such as patients. The technical developments described in our last publication in 2019 (2) have supported continued extensive growth of the BioSamples database, a variety of new user communities and use cases, and allowed us to quickly address emerging needs such as those driven by the SARS-CoV-2 pandemic. This has led to great additions in terms of content; BioSamples' size has more than tripled in 3 years from 5 to 18 million samples. With this growth in scale and diversity, though, comes a challenge of harmonisation. In the life sciences, there is a diverse and heterogeneous range of communities practicing open science and taking advantage of established research infrastructures such as ELIXIR core data and deposition resources (3). This diversity is reflected in the content of the BioSamples database. There are currently over 50 000 unique attributes and 60 million unique attribute values in BioSamples; and over 24 different attributes containing longitude related information for example. Sample collection and data generation is an expensive undertaking, leading to an increasing desire to combine and reuse datasets from the public domain, integrating multiple data modalities to generate larger sample sizes and enable higher power analyses (4,5). In this paper, we highlight the value provided by the BioSamples database, supporting the global research community to address some of these data integration challenges.

**Figure 1.**
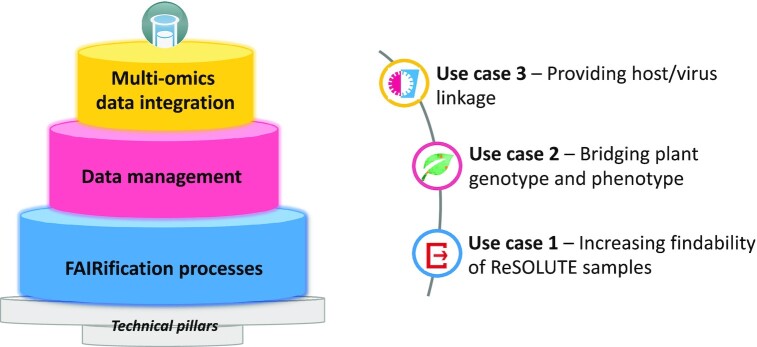
The BioSamples layer cake of FAIRification. Technical pillars underpin three levels of data management supporting different communities of users described in subsequent sections.

Building on the renewed technical foundations described in our last NAR publication (2) the BioSamples database now provides three levels of sample data management support as shown in Figure 1. At the lowest level, automated ‘FAIRification’ processes can be exploited to validate and semi-automatically curate sample records, which can be assimilated into any data management infrastructure. The ReSOLUTE use case describes a scenario that exploits this new functionality to enable greater findability of ReSOLUTE data assets. At the second level, data managers can rely on the BioSamples database for sample management, potentially entirely outsourcing the need for development of sample tracking databases. The plant use case section highlights how the plant omics community is relying on BioSamples as a foundation data deposition infrastructure to provide a common source of reference. Finally, at the highest level, BioSamples provides interconnectivity across assay data archives, and we describe a SARS-CoV-2 use case that explains how genotyping data from patients with COVID-19 can be combined and cross-referenced with datasets taken from viral isolates of samples from those patients, exploring the pathogenicity of SARS-CoV-2 and its potential interactions in the host. Finally, we outline some common technical pillars that the BioSamples database has established that can be combined to exploit value specific to new use cases and communities, for example by providing automated refinement and harmonization of clinical cohort data dictionaries, or through the adoption of common processes recommended by the FAIRplus cookbook or IMI-sponsored data management plans.

## SUPPORTING THE OMICS COMMUNITY

The BioSamples database has worked closely with many groups and communities to provide support for combining datasets from shared or similar biological samples. Below, we describe three exemplar use cases that exploit the scenarios supported by the BioSamples database, as shown in Figure 1.

### ReSOLUTE use case: FAIR improvements for cell line transcriptomics

Solute carrier (SLC) proteins facilitate the transport of substance across biological membranes, and play important roles in physiological processes involved in disease development and progression (6). The Research Empowerment on Solute Carriers (ReSOLUTE, https://re-solute.eu) project is a public-private partnership between academia and industry which aims to increase studies on SLCs for medical research and development. In one ReSOLUTE study, seven human cell lines were used to generate solute carrier (SLC) knockouts and overexpressing cell clones, which were then assayed to explore the transcriptional profile. Whilst the resulting dataset was publicly shared and made available in the SRA archive, additional information about the cell culture, cell line description was only available in a separate PDF file, making it hard to leverage this information efficiently. To support greater discoverability of ReSOLUTE datasets, curators for ReSOLUTE were able to develop extract, transform and load (ETL) procedures that mined experimental details from the bespoke, study specific PDF files and added details about cell cultures through the BioSamples curation API, https://www.ebi.ac.uk/biosamples/docs/guides/curation. This API allows both data owners and data users to curate samples, making it easier to correct errors or integrate additional information in the metadata. Those curation objects are added as extra layers to the metadata, capturing the curator ID and curation date, which allows retrieval of metadata at a specific time point for provenance tracking. An uncurated view of the data set is also available.

To ensure the curated ReSOLUTE metadata meets broader community requirements and to make the publicly accessible transcriptomics datasets more reusable, BioSamples metadata checklists validation services make it simple to validate metadata on those ReSOLUTE cell line used in transcriptomics assays. The Minimum Information about a high-throughput Nucleotide Sequencing Experiment (MINSEQE) standard (7) provides information about what metadata needs to be collected for transcriptomics samples, study and projects; it has been used by the Gene Expression Omnibus (GEO) (8) and ArrayExpress (9) databases. BioSamples provides a JSON schema representation of many standards, including MINSEQE to support automated validation. Newly curated ReSOLUTE samples were automatically validated against the MINSEQE checklist, ensuring sample records are FAIR at the point of deposition. Besides adding metadata from the project description, linking the metadata to external knowledge-base provides more detailed and accurate information for data interpretation. For example, the Cellosaurus database (10) collects rich metadata of cell lines, with detailed description, cross-reference, as well as publication citations. To standardise and consistently represent the property of cell lines used in the Resolute project, the corresponding Cellosaurus IDs were added to the sample metadata, linking sample entry in the BioSamples data archive to cell line description in Cellosaurus.

With the improvements provided via BioSamples curation APIs, the ReSOLUTE sample metadata are annotated with more detailed information, enriched with ontology terms enabling ontology-based search, and can be downloaded in standard formats such as JSON and XML for downstream automated analysis. Availability: ReSOLUTE samples can be accessed at https://www.ebi.ac.uk/biosamples/samples?filter=attr:project:RESOLUTE.

### Plant use case: bridging genotype to phenotype

Currently, most plant genotypic and phenotypic datasets are disconnected, making it hard to elucidate relationships between both, which is fundamental for accelerated crop breeding. Development of high-throughput and high-resolution technologies have led to an increased number of plant phenotypic datasets being generated, with higher complexity and of larger size (11). Reuse of phenotypic data is not only challenging due to its heterogeneity, but also due to the need of integrating genotypic and phenotypic data (11,12). Phenotypic information is fragmented across institutional repositories, and genotypic information is at best not linked, at worst completely absent from public archives such as the European Variation Archive (EVA, 28). This decreases findability of datasets, their interoperability and therefore their reusability. Indeed, apart from the obvious categories (authors, project, laboratories, technologies used, file format) it is critical to ensure the quality of key attributes, including plant material identification, genome assembly version, computational tools and environments used to generate the data. For plant phenotyping data, BioSamples collaborates with the FONDUE project, https://elixir-europe.org/about-us/commissioned-services/fondue, implements the Minimum Information About a Plant Phenotyping Experiment (MIAPPE) (11) standards as a sample checklist, and links plant study data in European Nucleotide Archive (ENA) (13) and EVA.

Ensuring data and metadata compliance with community standards requires good data validation tools; we have deployed an enhanced submission flow of genotypic data to archives like EVA or ENA. To enable validation, users can select their checklist of choice—such as MIAPPE—when uploading data to BioSamples. To store those checklists a new JSON Schema store was developed recently as part of the Global Alliance for Genomics and Health (GA4GH) (14). In addition to storing checklists encoded as JSON Schema, the JSON Schema store promotes best practices of checklist usage by encouraging reuse, improving checklist discovery, and having a consistent mechanism for versioning and schema retrieval thereby improving reproducibility. The JSON Schema store currently hosts both archive specific checklists such as ENA, and community driven checklists such as MIAPPE, which reflects that the quality of the metadata is a collective responsibility. Additionally, to ease submission and complement the structured format we developed to support complex phenotypic features description presented in our previous paper, we are now supporting submissions in the ISA-tab format (15) through a spreadsheet template and a ‘drag’n’drop’ uploader where files containing samples metadata can be uploaded and the samples are validated and persisted to BioSamples. Finally, to streamline integration with the EMBL-EBI archives hosting plant metadata and improve the provenance tracing of the data, we have harmonized the authentication processes between BioSamples, ENA and EVA, and now support authentication through the ENA Webin Authentication Service (16). This means users wanting to deposit data across those archives only need one set of credentials throughout, they retain ownership of their data across repositories and they can update and modify it directly in the right archive. Availability: An example of plant sample submitted by FONDUE is available at https://www.ebi.ac.uk/biosamples/samples/SAMEA104614465.

### COVID-19 use case: linking patient and pathogen samples to study the impact of host and virus genetic variability

Over the last 18 months, the COVID-19 pandemic has highlighted challenges in sharing sample and assay data globally that is both harmonized well enough and disseminated quickly enough to allow reuse and meta-analysis across groups. Understanding host and environment-specific SARS-CoV-2 infection rate and COVID-19 severity requires better tracking of the relationship between host, virus and environmental samples (17,18). As an established central hub for biological sample metadata, many COVID-19 viral sequenced sample records were deposited in BioSamples from early in the pandemic and from a wide range of data sources, including direct sample submissions, imported samples from the ENA and exchanged sample records from the NCBI BioSamples database (19,20). Consequently, BioSamples provides the basis platform for collective storage and on demand retrieval of COVID-19 samples (samples related to SARS-CoV-2 exposure or COVID-19 disease) metadata for the COVID-19 data portal (21). The COVID-19 portal brings together relevant datasets submitted to EMBL-EBI and other major centres for biomedical data with the aim to facilitate data sharing and analysis, and to accelerate coronavirus research. As of September 2021, the COVID-19 data portal has indexed over 1.2 million COVID-19 samples from BioSamples.

Due to lack of standard nomenclature for COVID-19 disease, virus, and sample collection methods, samples collected during early stages of the pandemic used different terminologies to describe and define the metadata, making it hard to harvest all COVID-19 samples. For example, COVID-19 related samples collected in the early stage had various labels including novel coronavirus pneumonia, nCoV pneumonia, Wuhan seafood market pneumonia virus, Coronavirus infected disease-19, etc. To support needs of the European COVID Data Portal, we used BioSamples curation APIs to manually curate COVID-19 related samples, in particular with respect to taxonomy—all SARS-Cov-2 samples records in BioSamples are now consistently annotated with the NCBI Taxonomy (22) ID NCBITaxon_2697049. Further, all human sample records for COVID-19 patients were consistently annotated with the MONDO_0100096 term when it became available, as shown in Table 1. To make this information available to the rest of the scientific community, these annotations have been added to the ZOOMA (https://www.ebi.ac.uk/spot/zooma/) knowledgebase. ZOOMA suggests ontology annotations based on evidence from other curators and databases and is utilised in automatic ontology annotation pipelines provided for all sample records in the BioSamples database. Curators and users can also provide their own ontology annotations. Such user/curator-provided annotation usually takes the sample attribute type and context into consideration and is manually reviewed. To maximize the high-confidence manual curation, we select manual ontology annotations and provide them as curation evidence to ZOOMA, which not only expands the ZOOMA curation database and facilitates further BioSamples annotation, but also allows those curations to be shared and reused by other projects and communities, contributing to increased data quality of the BioSamples database and other resources. For example, when running ZOOMA on our samples, we noticed a different entry from the Gene Expression Atlas (GXA) (19) annotating SARS-COV-2 to the syndrome rather than the virus: we liaised with their helpdesk and this was promptly resolved by updating corresponding GXA entries.

**Table 1.** Manual curation applied to specific samples and exported to ZOOMA. All future samples with similar attribute values will be annotated automatically through the ZOOMA pipeline

BioSamples ID	Attribute name	Attribute value	Ontology annotation
SAMN14168014	strain	SARS-CoV-2	http://purl.obolibrary.org/obo/NCBITaxon_2697049
SAMN14450688	host disease	COVID19	http://purl.obolibrary.org/obo/MONDO_0100096
SAMN14428242	isolation source	nasal swab	http://purl.obolibrary.org/obo/NCIT_C155833

To further support the COVID portal and research, the submission of samples metadata to BioSamples needed streamlining to allow for the unprecedented scale of the data flow, in particular for researchers from smaller laboratories depositing data manually. We developed and deployed the COVID-19 drag-and-drop data submission tool, embedded in the COVID-19 data portal. The uploader provides a ‘one-stop shop’ where data and metadata files are selected and dragged into the upload window in a single step and brokering takes place behind the scenes on behalf of the users. After the metadata files are parsed, the sample metadata is directly submitted to BioSamples, and the sequencing information is brokered to the ENA. Availability: COVID related samples can be accessed at https://www.ebi.ac.uk/biosamples/samples?text=NCBITaxon_2697049.

## FAIR AT THE FIRST MILE

The growth in number of user communities also reflects the outreach efforts towards promoting pre-accessioning of samples to improve consistency and comprehensiveness of records—such as with Tara Oceans (23) or biobanks who routinely create unique identifiers for samples to be collected or provided to users. In those projects, samples are often collected well in advance of their use in assays, and by the time assay data is ready for submission to public repositories, much metadata sample may otherwise have been lost, discarded, or inaccessible to the data depositor. Early registration of samples enables the original material provider to ensure each sample is consistently described according to their best practices such as MIABIS standard (24), as well as providing a unique ID to track samples and assays performed on them across archives and time. This promotes increased findability and accessibility of samples metadata through archives and time: a sample ID created by a biobank is passed on to the scientists when they acquire the sample. Its metadata remains consistent and validated but can also be extended and linked to new results from new assays in different archives.

To increase data quality as required by the use cases above and the scientific community in general, we have leveraged the wealth of data in BioSamples to deploy machine learning techniques to semi-automatically curate samples and provide content recommendation based on their similarity. As part of the GA4GH consortium, we have manually complemented those automated curations to target specific domains of interest, such as human disease.

### Improved data quality in public archives

To reduce the breadth of attributes that describe the same concepts across samples of similar types (e.g. how diseases are expressed in clinical samples), and therefore to increase the findability of samples relevant to a given search, we have developed the Curami tool, https://github.com/EBIBioSamples/curami-v2, to harmonise attributes in the BioSamples database. Curami promotes consistency between samples and underpins further processing such as described below for Phenopackets (http://phenopackets.org) export: standard representation of shared attributes across samples allows for consistent semantic annotation and export. Curami is an offline workflow which consists of multiple pipelines for data processing, analysis and integration. It analyses all existing attributes in the BioSamples database and processes them using machine learning and text mining to identify similar pairs of attributes that can be merged. Identified pairs can be merged automatically if above a confidence threshold or listed for a human review otherwise. Automatic and human assisted curations altogether were able to reduce the attribute space by 10% by merging >3000 different attributes. Altogether these 3000 attributes affected >30 million key value pairs and curated >10 million samples, aiming to improve the recall of the BioSamples search API. Consequently, these curations improved the recall of the BioSamples search API. For example, after curation, the search term ‘sample type’ yielded 25% more results (∼180 000 samples). Table 2 lists a few types of merged attributes.

**Table 2.** Merged attributes in curation process. Attribute 1 refers to the most popular or selected attribute. In the merging process attribute 2 will be replaced by attribute 1

Attribute 1	Attribute 2	Description
aluminium	Al	Short forms
disease	illness	Synonym
collection time	collection timestamp	
environmental feature	environmetal feature	Spelling mistakes
host disease status	host disease stautus	
cell surface marker	cell surface markers	inflections
samp size	sample size	
age years	age yrs	Different units and formatting

Based on the curations learned from Curami, we have developed a recommendation engine which is able to provide attribute suggestions for a given sample. The recommendation engine uses popular attributes in BioSamples and learned rules in Curami to suggest improvement to attributes. It is targeted towards data submitters without knowledge of existing community standards who tend to use customized and informal data representations. The service identifies attributes such as ‘collection date’ and ‘organism part’ and suggests spelling and format alterations. After correction, data records are more consistent resulting in increased findability.

To improve FAIRness of samples at the source, i.e., at submission time, and to encourage submission of quality data, we have introduced sample validation and certification services. The sample validation service is run interactively upon new submission and compares submitted data against a given checklist. It fails the submission upon error, such as when required fields are not present or when their values do not comply with the checklist's requirements. The sample certification service is an offline process which certifies each sample with a list of all the checklists they are compliant with, and additionally identifies samples which are ‘almost-compliant’ to any checklists known to BioSamples. This process provides vital information to data submitters for discovering similar data standards and identifying primary curation targets for FAIRer data. Both the validation and certification services rely on the ELIXIR biovalidator, https://github.com/elixir-europe/biovalidator, developed at EMBL-EBI. The ELIXIR biovalidator extends default JSON Schema validation vocabulary by introducing ontology validation. Therefore, the validation process ensures both syntactic and semantic validity of a sample. For example, the semantic validation process could ensure that the value of the ‘disease’ attribute can be constrained to being a subclass of the ontology term MONDO:00000001, disease.

**Figure 2.**
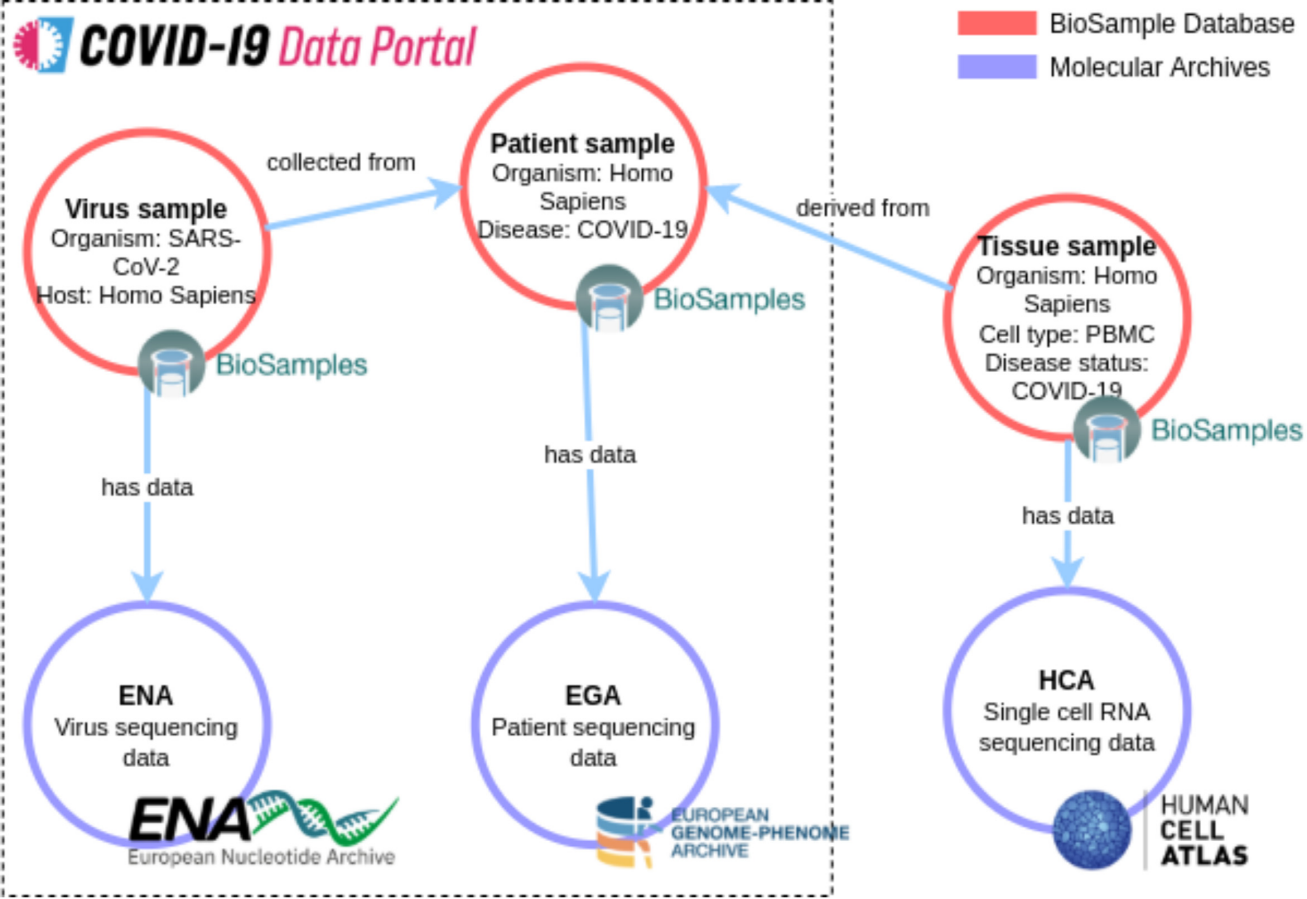
Sample relationships and inter-archival relationships. A donor patient sample (top middle) is hosted in EGA under controlled access for privacy and confidentiality. A tissue sample (top left) is generated from that donor and its information hosted in EGA as well. Both samples have had BioSamples IDs assigned upon submission. Metadata attributes that can be made public are then imported by BioSamples, where that metadata can be linked to the corresponding viral sample (top left) in BioSamples, which sequencing data is hosted by ENA and linked to the sample.

BioSamples is a hub connecting to other archives at EBI through explicit relationships, which enhance connectivity between samples and render a complex connected graph of sample and other resources. This knowledge graph enables cross archive resource discovery and helps to discover datasets which can otherwise only be found by manually, individually, querying multiple archives. To leverage existing sample connections and to enable complex relationship queries, we have developed a graph-based search service. The graph-based search uses Neo4j to index the relationships embedded in samples in the back end, while in the front-end the user interface allows to execute advanced relationship-based queries to discover cross-archives resources. This development was driven by the need to support association of viral samples in ENA to their human counterpart in EGA, and our early proof of concept demonstrated it was possible to provide queries to enable users to search across multiple archives, such as finding (‘covid-19 pathogen samples in ENA’ associated with ‘human samples in EGA’) as shown on Figure 2. This means users finding COVID viral samples in ENA will be able to link those samples, through their BioSamples IDs and relationships, to their human counterpart in the EGA.

### Expanding to other communities

As an ELIXIR Deposition Databases for Biomolecular Data, BioSamples focuses on the archiving and distribution of FAIR data and connects sample metadata with other data archives. For single cell studies, data in the Human Cell Atlas (HCA) (25) project are submitted to BioSamples and linked to sequencing data in ENA and the European Genome-phenome Archive (EGA) (26) through their sample accessions. BioSamples’ FAIR focused infrastructure supports features such as curation, ontology-powered search, checklist, and validation, all geared towards enriching metadata quality. Besides connecting with different biomedical communities, BioSamples also works with European projects such as FAIRplus, https://fairplus-project.eu/, where it leads activities aimed at making life science data FAIR by serving as a sample hub for sample metadata hosting and distribution, supporting the development of metadata models, and providing tools and guidelines for data curation and validation for both academic and industrial partners as described in the RESOLUTE use case above. This supports design and deployment of new software tools and best practices to not only enrich data in BioSamples but also allows the biomedical community to make the most of the data.

GA4GH aims at enabling responsible genomic data sharing for the benefit of human health. As of September 2021, BioSamples contains just under 2 million human samples (10%) associated with disease/phenotype information, and often linked with additional data in molecular archives. Phenopackets is the GA4GH standard to describe human disease and phenotypes, allowing biologists and clinicians to find, interpret, and develop complex models based on phenotypic data. However, the heterogeneity of the disease and phenotype metadata representation in samples was preventing its optimal inclusion in the Phenopackets we export. Therefore, to improve the phenopacket content with additional disease-related metadata, we infer disease information based on the existing metadata and add a curation with an additional attribute such as ‘BioSamples inferred disease: diabetes’, allowing the explicit inclusion of the disease in the phenopacket export. As of today, rules for 15 different disease types have been established, leading to the curation of 28 576 samples. Further disease types are currently being added, and disease ontology annotations will be exported to ZOOMA. The addition of automated curation tools to the existing cross-linking capabilities, coupled with the ability to support structured data types as described in (2) has made BioSamples an attractive proposition for further molecular archives such as PRIDE and Metabolights who are in the process of connecting with BioSamples APIs at submission time to natively support cross-referencing. In turn, this has contributed to technical developments on the BioSamples side, aimed at streamlining and scaling up submissions, while improving validation and enriching samples metadata. For example, we have deployed a new bulk accessioning API where multiple samples can be registered at once, instead of individual calls for each sample. Bulk accessioning straightaway decreases the network latency *n*-fold (n being the number of samples registered in one API call), supporting requests for increasing sizes of datasets. Similarly, and while we were already supporting the addition and presentation of antibiogram structured data as described in (2), in 2020 we introduced distinct access control and ownership to the structured data component in the overall samples metadata. This enabled the HoloFood project, which investigates impact of feed additives to animal-associated gut microbiome, to add structured data as curation objects to samples they did not originally submit, enriching them with laboratory data that couldn’t be included with the sequence deposition in ENA, and which would have otherwise been lost.

## DISCUSSION

Through different targeted efforts and projects, we are improving the data quality and FAIRness of samples metadata in BioSamples, impacting diverse communities of users at different levels of their FAIRification journey. This also entails a closer engagement with the community, and dynamic reprioritisation of work based on new, emerging requirements. For example the ability to run case insensitive search which has been recently reported will be addressed in an upcoming release as we are able to update the underlying indexing software. While we have dedicated resources to perform retrospective data quality improvement such as post-submission curation of samples metadata through automated pipelines, an important consideration is the ability to effect data management practices from the start of a new project. We believe this can also be successfully achieved by engaging early with communities of interest, such as demonstrated in the ReSOLUTE use case, where an initial overhead to adapt planned practices results in overall better, FAIRer metadata for all subsequent outputs of the project. During this work, emphasis has been put on maintaining and expanding shared technical capabilities we had released in anticipation and describe in (2). This will promote interoperability across projects and allow development of interoperable, modular and reusable products thereby improving sustainability prospects.

Those specific feature-targeted developments underpin and complement the submission FAIRification toolkit we are currently developing. Providing a FAIRification toolkit to external communities aligns with our vision of enabling federation rather than centralisation, making our tools available to users who seek to adopt best practices underpinning BioSamples. To address requirements for validation and compliance with user-provided checklists, we have deployed a JSON schema validator, the ELIXIR biovalidator, which in turns relies on the JSON schema store described earlier. In addition to consolidating well-established checklists in a common format across BioSamples and ENA, the JSON schema store enables users to add their own bespoke validation schema - this means specific consortia can require specific validation rules in addition to archives constraints at the same time and ensure consistency of the samples metadata with both sets of rules. For example, the PHA4GE consortium's SARS-CoV-2 checklist (27) has been added to the JSON schema store, and can be used in conjunction with the ERC000033 checklist (https://www.ebi.ac.uk/ena/browser/view/ERC000033) required by ENA and the COVID portal for pathogen sequence submission. Some planned addition to our FAIRification toolkit include adding a converter from JSON schemas to submission templates, making it easier for submitters to download the right template for the right version of the right checklist and ensuring compliance with guidelines. Sharing our toolkit also promotes interoperability of other tools and pipelines outside BioSamples, making it easier for users to adopt and removing unnecessary dependency on the BioSamples platform.

Making human data maximally useful and discoverable whilst respecting privacy concerns is a careful trade-off. Care must be taken when sharing metadata publicly to ensure no identifiable information is released. We are working with the EGA team to define a standard for non-identifiable human metadata, starting with the set of attributes that EGA already considers publicly releasable, i.e. ‘sex’, ‘donor ID’ and ‘phenotype’, and that are currently available through an EGA-provided public API. In the future we would like to deploy a framework in which those shared standards enable early pre-registration without compromising identifiability of the patient. BioSamples would become the central hub for discovery of human samples and enable virtual cohort construction, prior to access request to specific, controlled-access, datasets. Shared, consistent identification of donors through their BioSamples identifiers will additionally allow linking of private and public metadata by users with appropriate permissions.

## DATA AVAILABILITY

The BioSamples database is freely available at http://www.ebi.ac.uk/biosamples. Content is distributed under the EMBL-EBI Terms of Use available at https://www.ebi.ac.uk/about/terms-of-use. The BioSamples code is available at https://github.com/EBIBioSamples/biosamples-v4 and distributed under the Apache 2.0 license.
